# Antibacterial Activities, Phytochemical Screening and Metal Analysis of Medicinal Plants: Traditional Recipes Used against Diarrhea

**DOI:** 10.3390/antibiotics8040194

**Published:** 2019-10-24

**Authors:** Nasir Mahmood, Ruqia Nazir, Muslim Khan, Abdul Khaliq, Mohammad Adnan, Mohib Ullah, Hongyi Yang

**Affiliations:** 1 Key Laboratory of Saline-alkali Vegetation Ecology Restoration, Ministry of Education, College of Life Sciences, Northeast Forestry University, Harbin 150040, China; 2 Department of Chemistry, Kohat University of Science and Technology Kohat 26000, Khyber Pakhtunkhwa, Pakistan; 3 Department of Botanical and Environmental Sciences, Kohat University of Science and Technology, Kohat-26000, Khyber Pakhtunkhwa, Pakistan; 4 Key Laboratory of Functional Inorganic Material Chemistry, Ministry of Education. School of Chemistry and Material Science, Heilongjiang University, Harbin 150080, China

**Keywords:** phytochemicals, heavy metals, recipes, MIC, *E-coli*, *Salmonella*, *Shigella and MRSA*

## Abstract

The aim of this study was to explore the phytochemical composition, heavy metals analysis and the antibacterial activity of six medicinal plants i.e., *Terminalia chebula* Retz (fruits), *Aegle marmelos* L., (fruits), *Curcuma longa* L., (rhizomes), *Syzygium aromaticum* L., (flower buds), *Piper nigrum* L., (seeds), *Cinnamomum cassia* L., (barks) and its two remedial recipes (recipe 1 and 2) used against diarrhea obtained from the local herbal practitioners (Hakeems). A preliminary phytochemical screening of the above-mentioned plants extract in methanol, chloroform, n-hexane and distilled water revealed the presence of various constituents such as alkaloids, flavonoids, tannins and saponins by using standard procedures. The quantitative phytochemical studies shows that alkaloids, flavonoid and saponins were in maximum amount in *Terminalia chebula*. The concentration of Cd, Ni, Pb, Fe, Cr, Cu and Zn were investigated by using an atomic absorption spectrometer. The obtained analysis shows that Cr, Fe and Pb were present in the highest concentration in medicinal plants and their recipes. The antibacterial activities of the crude extract found in the recipes of methanol, chloroform, n-hexane and distilled water were analyzed by using agar well disc diffusion assay and minimum inhibitory concentration (MIC) by broth dilution method against four bacterial strains, namely, *E. coli, Salmonella, Shigella* and *Methicillin-resistant Staphylococcus aureus (MRSA)*, respectively. The maximum zones of inhibition in methanol, water, chloroform and n-hexane extracts were seen in recipe 2 against *Shigella* (22.16 ± 0.47 mm), recipe 2 against *Shigella* (20.33 ± 0.24 mm), recipe 1 against *Shigella* (20.30 ± 0.29 mm) and recipe 2 against *E. coli* (30.23 ± 0.12 mm), respectively. Furthermore, the recipe extracts are more active against the tested bacterial strains than the extracts from individual plants. Therefore, it is concluded that the use of herbal plants and their recipes are the major source of drugs in a traditional medicinal system to cure different diseases.

## 1. Introduction

The present study was conducted in district Karak, in the southern region of Khyber Pakhtunkhwa, Pakistan. The total population of district Karak is 706,299, with an area of about 3372 square Kms. Karak is situated between 31°15′ and 36°55′ latitude and between 70°05′ and 74°05′ longitude. Most of the people live in rural areas and depend on medicinal plants for the curing of different diseases. Medicinal plants can act as an indigenous source of new compounds possessing therapeutic value and can also be used in drugs development. According to the World Health Organization (WHO), plants can provide different varieties of drugs for low-income nations to cope with their primary healthcare needs [1]. The plants are medicinally important due to the presence of biologically active secondary metabolites such as alkaloids, flavonoids, steroids, saponins and terpenoids, which exert their effects by interacting with human physiology. The antimicrobial activities of these phytochemicals are due to their chemical nature [2,3] and are a potential source of diarrheal disease [4]. For this reason, the WHO has encouraged the studies for the treatment and prevention of diarrheal diseases using traditional medicinal practices [5]. Presently, a large number of medicinal plants are being used in many countries of the world, including Pakistan, due to their anti-diarrheal properties [6]. In Bangladesh, over 250 floral species are used by the folk medicinal and tribal healers for the treatment of diarrhea [7]. The Indian Himalayan region also support approximately 1700 plant species of known medicinal value [8]. However, there are only few studies on the utility of medicinal plants in the treatment of specific diseases [9]. Various medications are used for diarrheal diseases which possess different adverse effects like nausea, headache, dry mouth and constipation. However, there are many medicinal plants that have anti-diarrheal activities with less, or even no, side-effects than the allopathic drugs. These plants show anti-diarrheal activity by reducing secretions and the gastrointestinal motility [10].

The medicinal plants are destroyed and contaminated by various factors, such as environmental pollution, soil harvesting, microbial growth and introduction of toxic metals. The ingredients of plants include metal ions which are responsible for nutritional as well as medicinal usage [11]. Heavy metals like zinc, manganese, cobalt, iron, copper, chromium and nickel are essential for proper body function and become toxic when they exceed the recommended level and cause various chronic and acute effects in the living organisms [12]; whereas, the metals like lead, mercury and cadmium are non-essential and are toxic in nature even in the trace amount [13].

Thus, due to the hazardous affects as well as antibiotic resistance to the synthetic drugs, researchers are trying to obtain the antimicrobial drugs from medicinal plants due to their non-toxic nature and less side effects [14,15]. In spite of all the progress in the field of allopathic drugs, the traditional medicines, particularly plant-based medicines, also have a key role. Many studies have shown that crude extracts of medicinal plants as well as the pure bioactive components can act as good therapeutic agents [16,17].

Apart from the individual medicinal plants used in this study, we also analyzed the two recipes and their MIC. These recipes were prepared and named according to the traditional Hakeems. Recipe 1 is named Akseer-e-Pechesh and recipe 2 is named Taryaq-e-tabkhir Balghami, respectively. We observed that recipe 1 contains *T. chebula* (fruits), *A. marmelos* (fruits) and *C. longa* (rhizomes), which are mixed in the ratio of 1:1:2, respectively. It is used in the form of powder which is relatively more effective against diarrhea and dysentery. Recipe 2 also contains *S. aromaticum (flower buds)*, *P. nigrum (seeds)* and *C*. *cassia* (barks), which are mixed in the ratio of 1:0.5:1 and ground to powder, which is also quite effective in the treatment of diarrhea and constipation. These plants, as well as their recipes, are substantially used by the local inhabitants and local herbal practitioner (Hakeems) to treat diarrhea caused by the pathogenic microorganisms like *E. coli, Salmonella, Shigella and MRSA.*

## 2. Results

Qualitative phytochemical screening shows that the alkaloids were present in chloroform and methanol extracts of *T. chebula* (fruits), *A. marmelos* (fruits), *S. aromaticum* (flower buds), *C. longa* (rhizomes), *C. cassia* (barks), *P. nigrum* (seeds), recipe 1 and recipe 2, while it was not detected in n-hexane extract of *A. marmelos* (fruits) and in aqueous extracts of *C. longa* (rhizomes), as shown in Table 1. Whereas, the quantitative amount of alkaloids determined in *T. chebula* (fruits), *A. marmelos* (fruits), *C. longa* (rhizomes), *S. aromaticum* (flower buds), *P. nigrum* (seeds), *C. cassia* (barks), recipe 1 and recipe 2 were 27.84%, 2.54%, 2.66%, 11.88%, 5.06%, 5.28%, 19.66% and 17.78% respectively, as predicted in Table 2. The Flavonoids are present in methanol extract of *A. marmelos* (fruits), *T. chebula* (fruits), *S. aromaticum* (flower buds), recipe 1 and recipe 2, while they were not detected in *P. nigrum (seeds), C. longa* (rhizomes) and *C. cassia* (barks). However, in the aqueous solution and chloroform extracts, the flavonoids were found in *A. marmelos* (fruits) and in recipe 1, while in n-hexane extract the flavonoids were found in *S. aromaticum* and in recipe 2, as shown in Table 1. The quantity of flavonoids in *T. chebula* (fruits), *A. marmelos* (fruits), *C. longa* (rhizomes), *S. aromaticum* (flower buds), *P. nigrum* (seeds), *C. cassia* (barks), recipe 1 and recipe 2 were found to be 61.21%, 23.81%, 6.82%, 18.6%, 9%, 4.74%, 28.13% and 14.25% respectively, as shown in Table 2. Saponins were found preliminary in methanol, aqueous, chloroform and n-hexane extracts of all the medicinal plants and their recipes, as shown in Table 1. Quantitatively, the amount of saponins were determined in *T. chebula* (fruits), *A. marmelos* (fruits), *C. longa* (rhizomes), *S. aromaticum* (flower buds), *P. nigrum* (seeds), *C. cassia* (barks), recipe 1 and recipe 2 to be 6.32%, 0.24%, 0.36%, 1.10%, 0.19%, 0.23%, 1.19%, and 0.83% respectively, as shown in Table 2. Tannins were detected preliminarily in all the parts of plants as well as their recipes except *C. longa* (rhizomes) in crude methanol extracts. Tannins were also absent in the aqueous extracts of *C. cassia* (barks) and *C. longa* (rhizomes), while in the case of crude chloroform extracts, it was undetected in *P. nigrum* (seeds), *C. longa* (rhizomes) and *C. cassia* (barks). In the n-hexane crude extracts it was found in *S. aromaticum* (flower buds) and in recipe 2, as shown in Table 1. The results obtained in this study are highly consistent with the previously reported results from the study of medicinal plants of this region [18,19,20,21,22,23].

**Figure 1:**
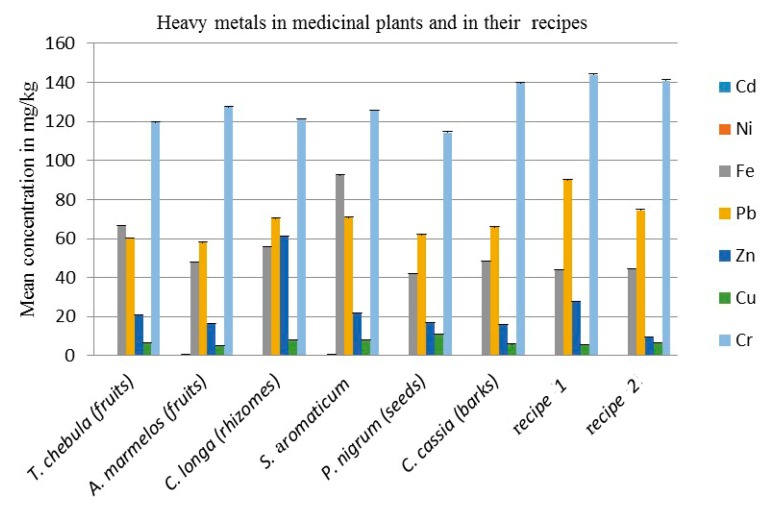
Mean concentration of heavy metals in mg/kg in medicinal plants and in their recipes. Each column represents the mean value of three independent replicates and the error bars indicate standard deviation.

The results of seven different elements shown in Table 3 and Figure 1 indicate that there is no cadmium at all. The cadmium (Cd) is very toxic, non-essential and the accumulation of cadmium may damage the kidneys. According to the WHO, the recommended level of Cd is 0.3 mg/kg in medicinal plants [24]. The results show that in *T. chebula*, *C. cassia*, recipe 1 and recipe 2, the Ni is below the detection limit. The *S. aromaticum* (flower buds) contain 0.825 mg/kg, *A. marmelos* (fruits) 0.55 mg/kg, *P. nigrum* (seeds) 0.35 mg/kg and *C. longa* (rhizomes) 0.05 mg/kg for the mean concentration of Ni respectively, which are below the standard recommended level. According to the WHO, the maximum permissible limit of Nickel in medicinal plants is 1.5 mg/kg, while its recommended level for mankind is 1 mg/day [24]. The results illustrate that the maximum amount of iron present in the *S. aromaticum* (flower buds) 92.45 mg/kg, *T. chebula* (fruits) 66.775 mg/kg, *C. longa* (rhizomes) 55.9 mg/kg, *C. cassia* (barks) 48.475 mg/kg, *A. marmelos* (fruits) 48.1 mg/kg, recipe 2 44.475, recipe 1, 43.875 mg/kg and *P. nigrum* (seeds) 41.975 mg/kg, is beyond the maximum permissible value. According to the WHO, the maximum permissible limit of iron in medicinal plants is 20 mg/kg, while its daily requirement is 10 to 28 mg/day [20]. The results further show that the high concentration of Pb was found in recipe 1, 89.9 mg/kg, recipe 2, 74.45 mg/kg, *S. aromaticum* (flower buds) 70.675 mg/kg, *C. longa* (rhizomes) 70.1 mg/kg, *C. cassia* (barks) 65.875 mg/kg, *P. nigrum* (seeds) 61.925 mg/kg, *T. chebula* (fruits) 60.125 mg/kg and *A. marmelos* (fruits) 58 mg/kg, which are beyond the maximum permissible limit as recommended by the WHO. According to the WHO, the maximum permissible limit of Pb in medicinal plant is 10 mg/kg [24]. The results further predict that the concentration of Zn was found in *C. longa* (rhizomes) 61.375 mg/kg, recipe 1, 27.925 mg/kg, *S. aromaticum* (flower buds) 21.75 mg/kg, *T. chebula* (fruits) 21.075 mg/kg, *P. nigrum* (seeds) 16.9 mg/kg *A. marmelos* (fruits) 16.325 mg/kg, *C. cassia* (barks) 16.025 mg/kg and recipe 2 9.475 mg/kg, which are below the maximum permissible level, except for *C. longa* (rhizomes), as permitted by the WHO. According to the WHO, the maximum permissible limit of Zn in medicinal plants is 50 mg/kg, while its daily requirement in food is 11 mg/kg [25]. The results in Table 3 reveal that the concentration of Cu was found in *P. nigrum* (seeds) 10.9 mg/kg, *S. aromaticum* (flower buds) 8.3 mg/kg, *C. longa* (rhizomes) 8.225 mg/kg, *T. chebula* (fruits) 6.8 mg/kg, recipe 2 6.557 mg/kg, *C. cassia* (barks) 5.875 mg/kg, recipe 1 5.675 mg/kg and *A. marmelos* (fruits) 5.175 mg/kg, which is below or according to the maximum permissible level. According to the WHO, in medicinal plants, the maximum permissible amount of Cu is 10 mg/kg, while its daily requirement in food is 2–3 mg/day [24]. The results also indicated that the amount of Cr in recipe 1 was 143.9 mg/kg, recipe 2, 140.926 mg/kg, *C. cassia* (barks), 139.65 mg/kg, *A. marmelos* (fruits), 127.375 mg/kg, *S. aromaticum* (flower buds), 125.6 mg/kg, *C. longa* (rhizomes), 121.05 mg/kg, *T. chebula* (fruits), 119.475 mg/kg and *P. nigrum* (seeds), 114.325 mg/kg. The amount of Cr which exists in all these plant parts as well as their recipe is above the maximum permissible value. According to the WHO, the maximum permissible level of chromium in medicinal plants is 1.5 mg/kg and its daily requirement is 0.2 mg [24].

**Figure 2:**
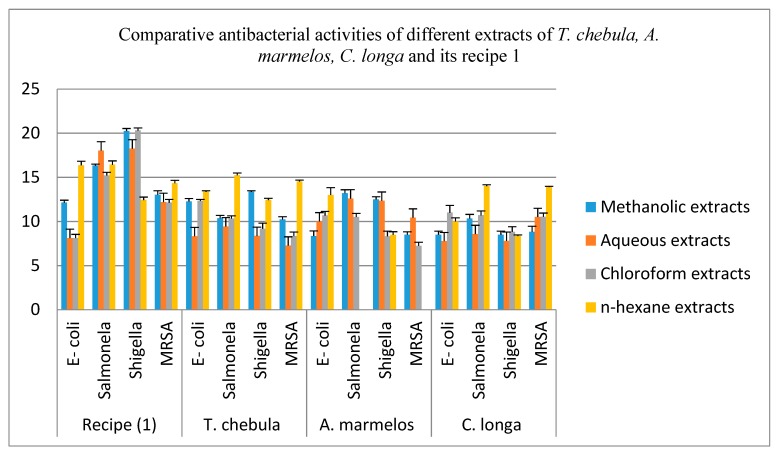
Comparative Antibacterial Activities of methanol, Aqueous, chloroform and n-hexane extracts of the *T. chebula* (fruits), *A. marmelos* (fruits), *C. longa* (rhizomes) and their recipe 1. Each column represents the mean value of three independent replicates and the error bars indicate standard deviation.

The comparative antibacterial activities of aqueous, methanol, chloroform and n-hexane extracts of the *T. chebula* (fruits), *A. marmelos* (fruits), *C. longa* (rhizomes) and their recipe 1 are shown in Table 4 and Figure 2. The aqueous extracts of *A. marmelos* shows very good inhibition effects against all four bacterial strains. The aqueous extracts of *T. chebula* (fruits) and *C. longa* (rhizomes) displayed a significant zone of inhibition against *Salmonella* and *MRSA* respectively which possess moderate inhibitory effects against the remaining bacterial strains. Recipe 1 presents a very good inhibition zone as compared to its individual plant parts against all bacterial strains except *A*. *marmelos*, which produced a large inhibition zone against *E-coli* only.

The methanol extracts of *T. chebula* (fruits) displayed a considerable inhibitory zone against all four bacterial strains. *A. marmelos* (fruits) and *C. longa* (rhizomes) also exhibit a strong inhibitory result except for *E-coli* and *MRSA*. Besides this, the *C. longa* also exhibited the moderate inhibitory effect against *Shigella.* Recipe 1 also revealed a very good inhibition zone as compared to individual plant parts against all four bacterial strains.

The chloroform extracts of *T. chebula, A. marmelos* and *C. longa* present good inhibition effects against *E-coli* and *Salmonella*. All these extracts exhibited a moderate inhibition zone against *Shigella*. The extract of *C. longa* shows a strong inhibitory effect against *MRSA,* while *T. chebula* and *A. marmelos* extracts show a moderate inhibition zone against *MRSA*. Recipe 1 revealed a significant inhibition effect against three bacterial strains, except for *E-coli,* as compared to its individual plant extract.

The n-hexane extracts of *T. chebula, A. marmelos* and *C. longa* displayed a marked effect against all four bacterial strains except the n-hexane extract of *A. marmelos,* which shows no inhibitory zone against *Salmonella* and *MRSA*. Recipe 1 of n-hexane indicates the better inhibition effect against all four bacterial strains as compared to all of its individual plant extracts.

**Figure 3:**
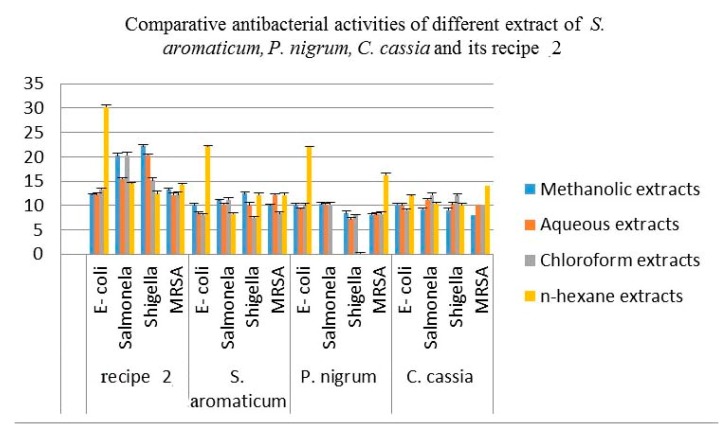
Antibacterial activities of methanol, aqueous chloroform and n-hexane extracts of the *S. aromaticum* (flower buds), *P. nigrum* (seeds), *C. cassia* and recipe 2. Each column represents the mean value of three independent replicates and the error bars indicate standard deviation.

The comparative antibacterial activities of aqueous, methanol, chloroform and n-hexane extracts of the *S. aromaticum* (flower buds), *P. nigrum* (seeds), *C. cassia* (barks) and their recipe 2 are shown in Table 5 and Figure 3. The aqueous extract of these three plants displayed a strong inhibition zone against *E-coli, Salmonella* and *MRSA*. Beside this, the *S. aromaticum* and *C. cassia* also show significant antibacterial activity against *Shigella* but the aqueous extract of *P. nigrum* indicated a moderate zone of inhibition against *Shigella*. The aqueous extract of recipe 2 revealed an excellent result, as compared to its individual plant extracts.

The methanol extracts of all these plants perceive very influential inhibitory effects against *E-coli* and *Salmonella*. The methanol extracts of these medicinal plants indicated a desirable inhibition zone against *Shigella* and *MRSA* except for the methanol extract of *P. nigrum,* which shows a reasonable zone of inhibition against *MRSA*. The methanol extracts of *C. cassia* also displayed a moderate zone of inhibition against *MRSA*. The methanol extract of recipe 2 shows a significant zone of inhibition against all the four bacterial strains as compared to its individual plant extracts.

The chloroform extracts of *S. aromaticum* displayed a significant inhibition effect against *Salmonella,* while a moderate inhibition effect against *Shigella, E-coli* and *MRSA*. The chloroform extracts of *P. nigrum* shows strong inhibition effects against *Salmonella* and *E-coli*, which demonstrated the moderate inhibition effects against *Shigella* and *MRSA*. The chloroform extract of *C. cassia* has strong inhibition effects against all four bacterial strains. The extracts of recipe 2 showed a very strong inhibitory effect, as compared to the individual plant parts against all four bacterial strains.

The n-hexane extract of *S. aromaticum* perceived more pronounced effects against *Shigella*, *E-coli* and *MRSA* and exhibited moderate effect against *Salmonella.* The extracts of *P. nigrum* demonstrated good inhibitory effects against *E-coli, MRSA* and show no zone of inhibition against *Shigella* and *Salmonella*. The *C. cassia* had a strong inhibition effect against all four bacterial strains. The n-hexane extracts of recipe 2 exhibited an appreciable inhibition zone as compared to its individual plants against all these four strains.

Minimum inhibitory concentrations (MIC) of recipe 1 and recipe 2 are shown in Table 6 and Table 7, respectively. Both of these recipes displayed minimum inhibitory concentrations (MIC) values against different bacterial strains. Recipe 1 shows MIC in methanol extract against *E-coli, Salmonella, Shigella* and *MRSA* at 11,000 mg/L, 12,000 mg/L 11,500 mg/L and 14,000 mg/L respectively, and similarly, in aqueous extracts against *E-coli, Salmonella, Shigella* and *MRSA* at 12,500 mg/L, 11500 mg/L, 12,000 mg/L and 14,500 mg/L, respectively. It shows MIC in chloroform extract against *E-coli, Salmonella, Shigella* and *MRSA* at 14,000 mg/L, 13,000 mg/L 14,000 mg/L and 15,000 mg/L respectively, and further, in n-hexane extract against *E-coli, Salmonella, Shigella* and *MRSA* at 15,000 mg/L, 15,000 mg/L 14,500 mg/L and 15,000 mg/L, respectively.

Recipe 2 displayed MIC in methanol extract against *E-coli, Salmonella, Shigella* and *MRSA* at 12,000 mg/L, 10,500 mg/L 11,500 mg/L and 15,000 mg/L, respectively. Similarly, it displayed MIC in aqueous extracts against *E-coli*, *Salmonella, Shigella* and *MRSA* at 11,000 mg/L, 12,000 mg/L 12,500 mg/L and 14,000 mg/L, respectively. It displayed MIC in chloroform extract against *E-coli, Salmonella, Shigella* and *MRSA* at 13,000 mg/L, 12,500 mg/L 13,000 mg/L and 15,000 mg/L respectively, and in n-hexane extract against *E-coli, Salmonella, Shigella* and *MRSA* at 14,000 mg/L, 13,000 mg/L 14,000 mg/L and 15,000 mg/L, respectively.

## 3. Discussion

Medicinally important plants and their biologically active phytoconstituents are used globally for curing various human diseases including gastrointestinal infections, inflammation, heart disease, cancer and respiratory infection. The use of herbal products and their recipes have fewer side effects as compared to synthetic drugs. All over the world, mostly, people depend upon the herbal products for their healthcare needs. Phytochemicals are the real sources of medicinal value of plants which exert their effects by interacting with human physiology [26]. Preliminary phytochemical screening was performed to find the presence of alkaloids, saponins, flavonoids and tannins. Alkaloids vary greatly in chemical composition and play a vital role in drugs discovery. Typically, the antimicrobial properties of medicinal plants were found due to alkaloids. Typically, herbal species containing flavonoids are known to have therapeutic properties and a declining ratio of cancer has been reported by consuming fruits and vegetables containing flavonoids [27,28]. Saponins are very effective in gastrointestinal infection and have antitumor properties [29]. Tannins have antibacterial activity and are used against diarrhea and dysentery. Tannins play a very important role in the healing of wounds and in bleeding [30]. Many studies have reported the clinical importance of the medicinal plants based on their phytochemical screening, which greatly reinforces the idea of novelty in research in this area [31,32].

Human beings use different medicinal plants from the time immemorial in many aspects, as nutritional values, remedy for different diseases and as essential components in cosmetics. The usefulness of these plants as well as the toxicity is due to their chemical nature, particularly due to the presence of heavy metals like zinc, manganese, cobalt, iron, copper, chromium and nickel. The cadmium (Cd) is very toxic and non-essential and the accumulation of cadmium damages kidneys and liver. Nickel (Ni) is an essential element for all living organisms and is required in a very minute quantity for an individual [33]. Above the permissible level, it is toxic and causes heart failure, loss of vision, loss of body weight and skin irritation [34]. Iron (Fe) is an essential component of hemoglobin. Its deficiency causes nose bleeding, myocardial infractions and gastrointestinal infection [35]. Lead (Pb) is a non-essential trace heavy metal having no functions both in animals as well as in plants. High concentrations of lead causes oxidative stress, brain damage, colic, anemia, headaches and central nervous system disorders [36]. It accumulates in the spleen, kidney and liver through air (20%), food (65%) and water (15%). Zinc (Zn) is an essential trace heavy metal and plays an important role in various processes including bone formation, brain development, wound healing, normal growth and behavioral response. It is essential in protein as well as in DNA synthesis. It regulates structural and catalytic functions of different enzymes [37]. Copper (Cu) is an essential element for normal growth and development as well as for many enzymatic activities. The concentration of copper above the permissible level causes hair and skin discoloration, respiratory and some other lethal effects in human beings [38]. Its deficiency causes anemia and Wilson’s diseases [13]. Chromium (Cr) is very essential for the metabolism of glucose, cholesterol and fat. Its concentration above the permissible level is carcinogenic and toxic in nature. The toxicity of Cr intake may appear in the form of a stomach ulcer, skin rash, kidney damage, lung cancer and nose irritation. Its deficiency may lead to elevated body fat and disturbance in proteins, lipids and glucose metabolism [39].

Due to harmful effects as well as antibiotic resistance to the synthetic drugs, researchers are trying to obtain antimicrobial drugs from medicinal plants due to their non-toxic nature. In this study, the crude extracts of *Terminalia chebula* (fruits), *Aegle marmelos* (fruits) and *Curcuma longa* (rhizomes) *Syzygium aromaticum* (flower buds), *Piper nigrum* (seeds) *Cinnamomum cassia* (barks) recipe 1 and recipe 2 showed a very good zone of inhibition against all four tested bacterial strains.

MIC of recipe 1 and recipe 2 were determined by using the broth dilution method against *E-coli, Salmonella, Shigella* and *MRSA* as shown in Table 6 and Table 7, respectively. Both of these two recipes presented MIC against all the tested bacterial strains.

## 4. Materials and Methods

### 4.1. Collection of Medicinal Plants Parts and Their Identification

Medicinally effective parts of the selected plants including *Curcuma longa* L., (rhizomes), *Terminalia chebula* Retz., (fruits), *Aegle marmelos* L., (fruits), *Syzygium aromaticum* L., (flower buds), *Piper nigrum* L., (seeds) and *Cinnamomum cassia* L. (barks) were collected from the local herbal market (pansori shops) of Karak, Khyber Pakhtunkhwa. Plants parts were identified and used for further experimentation.

### 4.2. Plants Grinding and Recipe Formulation

The collected medicinal plant parts were initially washed using distilled water, dried and then sliced into small pieces, separately. Then, each part was mashed to form powder with the help of mortar and pestle, and these powdered samples were stored in a dirt-free separate closed glass container for further use. Apart from the individual plant parts, two recipes were also formed according to the Hakeem description [40,41,42]. Recipe 1 (Akseer-e-Pechesh) contains *T*. *chebula* (fruits), *A*. *marmelos* (fruits) and *C. longa* (rhizomes), which are mixed in the ratio of 1:1:2, respectively. Recipe 2 (Taryaq-e-tabkhir Balghami) contains *S. aromaticum (flower buds)*, *P. nigrum (seeds)* and *C. cassia* (barks), which are mixed in the ratio of 1:0.5:1, respectively.

### 4.3. Preparation of Plant Extracts

Extracts of each plant and its recipe were prepared by soaking 300 g of plant materials in 500 mL of four different solvents like methanol, distill water, chloroform and n-hexane. The mixtures were kept for 48 h stirring at room temperature, followed by the vacuum filtration. After that, the filtrate was rotary evaporated to obtain semi-solid extract. Then, the phytochemicals analysis in each sample were determined qualitatively and quantitatively [43,44].

### 4.4. Qualitative Phytochemical Screening

Qualitative phytochemical screening of medicinal plant parts and their recipes were carried out by means of some specific methods. Alkaloids, flavonoids, tannins and saponins were detected by the Tyler [45] and Harborne [46] method.

### 4.5. Quantitative Phytochemical Screening

Quantitative phytochemical analyses were carried by using the Harborne [46] and Obadoni [47] methods for the determination of alkaloids, the Boham [48] method for flavonoids and the Obadoni [47] method for saponins.

### 4.6. Heavy Metal Analysis of Medicinal Plants and Their Recipes

Heavy metals like Ni, Cd, Fe, Cr, Zn, Cu and Pb in the medicinal plants and its recipes were analyzed by an atomic absorption spectrophotometer (Perkin Elmer analyst 400, UK) using nitrous oxide (N_2_O)-acetylene flame. For the calibration of equipment, the following sensitivity and detection limits were established, in the amounts of: Ni (0.5, 1 and 2 ppm), Cd (0.5, 1 and 1.5 ppm), Fe (2, 4 and 6 ppm), Cr (2, 4 and 6 ppm), Zn (0.5, 1 and 1.5 ppm), Cu (2, 4 and 6 ppm) and Pb (2, 10 and 20 ppm).

A homogeneous mixture of hydrogen peroxide (H_2_O_2_) 30% and nitric acid (HNO_3_) 65% in 1:2 strength was prepared. One gram of each plant part in the form of dried powder was dissolved in this solution. The sample solutions were heated on a hot plate at 130 °C until the volume of each sample was reduced to 3 mL. Then, the solution was cool down, filtered and the volume was made up to 25 mL [49].

### 4.7. Antibacterial Activity

Collection of Bacterial strains and antibacterial activity.

Pure cultures of four bacterial strains e.g., *Shigella, Escherichia coli, Salmonella* and *MRSA* were obtained and selected for further experimentation. These four bacterial strains were further sub-cultured on nutrient agar. The agar well disc diffusion method was adopted for the evaluation of antibacterial activity. All the equipment was autoclaved and sterilized for 15 min at 120 °C before use. Then, 15 mL of the media was poured in each Petri plate and kept for cooling. The bacterial strains were applied on the Petri dishes by using a sterilized cotton swab. After that, by using the sterilized Cork borer of 6 mm diameter, each Petri plate was punched into five wells for DMSO, distilled water, chloroform, methanol and n-hexane crude extracts, respectively. After that, the stock solution of all crude extract in DMSO each of 30 mg/mL was prepared. Now, each well was filled with 100 µL stock solutions except one, which was filled with DMSO as a negative control. The standard disc of Ceproflaxacene (5 µg) was used as a positive control. All the processes were performed in the laminar flow hood in order to resist contamination. The plates were then incubated in the incubator at 37 °C for 24 h. At last, the zones of inhibition were measured in mm for each crude extract by using Digital Vernier Caliper and the obtained results were noted and recorded [50].

### 4.8. Determination of Minimum Inhibitory Concentrations (MICs) by Broth Dilution Method

MIC is the lowest concentration (in mg/L) of the antimicrobial agent that prevents visible growth of a microorganism under defined conditions. Broth dilution techniques (micro-dilution) were used to determine the minimal inhibitory concentration (MIC) of antimicrobial agents, including antibiotics that kill (bactericidal activity) or inhibit the growth (bacteriostatic activity) of bacteria. Broth dilution uses liquid growth medium containing geometrically increasing concentrations (typically a two-fold dilution series) of the antimicrobial agent, which is inoculated with a defined number of bacterial cells. The antibacterial agents were dissolved in DMSO. After incubation, the presence of turbidity or sediment indicates growth of the organism [51].

### 4.9. Statistical Analysis of the Data

The as-recorded data was analyzed and organized by means of Microsoft Excel. The entire experiments were performed three times, consecutively. Standard deviations as well as average zone of inhibitions were calculated by Microsoft Excel 2007. SPSS version 16 was applied to determine the phytoconstituent activities.

## 5. Conclusions

In summary, the overall results obtained show the medicinal values of the tested plant parts and their recipes, which are used against diarrhea. The qualitative phytochemical screening of these plants and their recipes exhibit the presence of alkaloids, flavonoids, saponins and tannins. The secondary metabolites are more potent in *T. chebula*, *S. aromaticum* and in their recipes. The atomic absorption study of the as-prepared plant samples predict the high concentration of Iron (Fe), Lead (Pb) and Chromium (Cr).

Therefore, it has been concluded that the present research work looking at the medicinal plants and their recipes used against diarrhea found that they are therapeutically active substances with enhanced activities. However, most of the crude extracts also show antibacterial activities. Furthermore, the recipes extracts are more active against the tested bacterial strains as compared to the extracts of individual plants. So, for this reason, the present research study is helpful to identify the bioactive compounds obtained from the medicinal plants which are used against different antimicrobial activities.

## Figures and Tables

**Table 1:** Qualitative phytochemical screening of alkaloids, flavonoids, saponins and tannins of medicinal plant parts and their recipes.

S. No	Plant Parts	Alkaloids				Flavonoids				Saponins				Tannins			
		Meth ext	Aqe Ext	Chlor Ext	n-hex ext	Meth Ext	Aqe Ext	Chlo ext	n-hex ext	Meth ext	Aqe ext	Chlor ext	n-hex ext	Meth Ext	Aqe ext	Chlor ext	n-hex ext
1	*T. chebula* (fruits)	+	+	+	+	+	−	−	−	+	+	+	+	+	+	+	−
2	*A. marmelos* (fruits)	+	+	+	−	+	+	+	−	+	+	+	+	+	+	+	−
3	*C. longa* (rhizomes)	+	−	+	+	−	−	−	−	+	+	+	+	−	−	−	−
4	*S. aromaticum*	+	+	+	+	+	−	−	+	+	+	+	+	+	+	+	+
5	*P. nigrum* (seeds)	+	+	+	+	−	−	−	−	+	+	+	+	+	+	−	−
6	*C. cassia* (barks)	+	+	+	+	−	−	−	−	+	+	+	+	+	−	−	−
7	Recipe (1)	+	+	+	+	+	+	+	−	+	+	+	+	+	+	+	−
8	Recipe (2)	+	+	+	+	+	−	−	+	+	+	+	+	+	+	+	+

Phytochemical detection key: (−) = Absent, (+) = Present; Meth = Methanol, Aqe = Aqueous, Chlor = Chloroform, n-hex = n-hexane, ext = extract.

**Table 2:** Quantitative phytochemical screening of alkaloids, flavonoids and saponins of medicinal plant parts and their recipes.

S. NO	Recipes/Plant Parts	Alkaloids %	Flavonoids %	Saponins %
1	Recipe (1)	19.66	28.13	1.19
2	*T. chebula (fruits)*	27.84	61.21	6.32
3	*A. marmelos (fruits)*	2.54	23.81	0.24
4	*C. longa (rhizomes)*	2.66	6.82	0.36
5	Recipe (2)	17.78	14.25	0.83
6	*S. aromaticum*	11.88	18.6	1.10
7	*P. nigrum (seeds)*	5.06	9	0.19
8	*C. cassia (barks)*	5.28	4.74	0.23

**Table 3:** Metals in herbal plants and their recipes e.g., Cd, Ni, Fe, Pb, Zn, Cu and Cr.

S. NO	Name of Plants Part	Cd mg/kg	Ni mg/kg	Fe mg/kg	Pb mg/kg	Zn mg/kg	Cu mg/kg	Cr mg/kg
1	*T.chebula (fruits)*	BDL	BDL	66.775 ± 0.016	60.125 ± 0.250	21.075 ± 0.013	6.8 ± 0.008	119.475 ± 0.430
2	*A. marmelos (fruits)*	BDL	0.55 ± 0.019	48.1 ± 0.002	58 ± 0.184	16.325 ± 0.004	5.175 ± 0.008	127.375 ± 0.347
3	*C. longa (rhizomes)*	BDL	0.05 ± 0.013	55.9 ± 0.072	70.1 ± 0.348	61.375 ± 0.003	8.225 ± 0.022	121.05 ± 0.099
4	*S. Aromaticum*	BDL	0.825 ± 0.005	92.45 ± 0.039	70.675 ± 0.401	21.75 ± 0.030	8.3 ± 0.003	125.6 ± 0.302
5	*P. nigrum (seeds)*	BDL	0.35 ± 0.045	41.975 ± 0.034	61.925 ± 0.150	16.9 ± 0.006	10.9 ± 0.015	114.325 ± 0.402
6	*C. cassia (barks)*	BDL	BDL	48.475 ± 0.075	65.875 ± 0.179	16.025 ± 0.040	5.875 ± 0.064	139.65 ± 0.467
7	Recipe (1)	BDL	BDL	43.875 ± 0.045	89.9 ± 0.449	27.925 ± 0.022	5.675 ± 0.020	143.9 ± 0.326
8	Recipe (2)	BDL	BDL	44.475 ± 0.077	74.45 ± 0.438	9.475 ± 0.064	6.557 ± 0.016	140.926 ± 0.314

Key: Values are mean ± standard deviation (SD), BDL: below detection limit.

**Table 4:** Comparative Antibacterial Activities of methanol, Aqueous, Chloroform and n-hexane extracts of the *T. chebula* (fruits), *A. marmelos* (fruits), *C. longa* (rhizomes) and their recipe 1.

Recipe/Plant Part	Bacteria	Methanol Extracts	Aqueous Extracts	Chloroform Extracts	n-Hexane Extracts	Cpx	DMSO
		(mm)	(mm)	(mm)	(mm)	Mm	(mm)
Recipe (1)	*E-coli*	12.13 ± 0.28	8.13 ± 0.44	8.13 ± 0.41	16.36 ± 0.47	32	–
	*Salmonella*	16.33 ± 0.17	18.03 ± 0.70	15.2 ± 0.37	16.43 ± 0.43	33	–
	*Shigella*	20.23 ± 0.30	18.26 ± 0.32	20.3 ± 0.29	12.43 ± 0.33	31	–––
	*MRSA*	13.03 ± 0.45	12.2 ± 0.24	12.13 ± 0.36	14.33 ± 0.32	23	–
*T. chebula* (fruits)	*E-coli*	12.26 ± 0.34	8.33 ± 0.48	12.33 ± 0.17	13.36 ± 0.12	32	–
	*Salmonella*	10.36 ± 0.33	9.43 ± 0.24	10.33 ± 0.30	15.23 ± 0.25	33	–
	*Shigella*	13.36 ± 0.12	8.36 ± 0.16	9.17 ± 0.63	12.43 ± 0.20	31	–––
	*MRSA*	10.23 ± 0.30	7.26 ± 0.26	8.34 ± 0.47	14.53 ± 0.16	23	–
*A. marmelos* (fruits)	*E-coli*	8.34 ± 0.58	10 ± 0.81	10.67 ± 0.48	13 ± 0.82	32	–
	*Salmonella*	13.2 ± 0.39	12.6 ± 0.43	10.5 ± 0.41	NIL	33	–
	*Shigella*	12.5 ± 0.29	12.34 ± 0.47	8.33 ± 0.57	8.47 ± 0.37	31	–––
	*MRSA*	8.5 ± 0.33	10.43 ± 0.42	7.24 ± 0.40	NIL	23	–
*C. longa* (rhizomes)	*E-coli*	8.5 ± 0.40	7.76 ± 0.20	11 ± 0.81	10 ± 0.41	32	–
	*Salmonella*	10.33 ± 0.47	8.57 ± 0.32	10.7 ± 0.49	14 ± 0.16	33	–
	*Shigella*	8.5 ± 0.40	7.8 ± 0.59	8.83 ± 0.57	8.36 ± 0.12	31	–––
	*MRSA*	8.83 ± 0.62	10.5 ± 0.24	10.53 ± 0.42	13.9 ± 0.08	23	–

Cpx = Ceproflaxacene; MRSA = Methicillin Resistant Staphylococcus Aureus; Noted ANNOVA value (*p* < 0.01) for all samples.

**Table 5:** Comparative Antibacterial evaluation of methanol, aqueous, chloroform and n-hexane extracts of the *S. aromaticum, P. nigrum, C. cassia* and recipe 2.

Recipe/Plant Part	Bacteria	Methanol Extracts	Aqueous Extracts	Chloroform Extracts	n-Hexane Extracts	Cpx	DMSO
		(mm)	(mm)	(mm)	(mm)	Mm	(mm)
recipe 2	*E-coli*	12.23 ± 0.49	12.26 ± 0.53	13.33 ± 0.12	24.23 ± 0.12	32	–
	*Salmonella*	20.23 ± 0.20	15.43 ± 0.33	20.33 ± 0.30	14.53 ± 0.44	33	–
	*Shigella*	22.16 ± 0.47	20.33 ± 0.24	15.16 ± 0.54	12.46 ± 0.26	31	–––
	*MRSA*	13.16 ± 0.41	12.13 ± 0.28	12.23 ± 0.46	14.23 ± 0.45	23	–
*S. aromaticum* (flower buds)	*E-coli*	10.06 ± 0.41	8.3 ± 0.37	8.03 ± 0.45	22.2 ± 0.20	32	–
	*Salmonella*	11.03 ± 0.36	10.2 ± 0.33	11.13 ± 0.25	8.23 ± 0.12	33	–
	*Shigella*	12.46 ± 0.25	10.1 ± 0.29	7.5 ± 0.40	12.1 ± 0.29	31	–––
	*MRSA*	10.03 ± 0.28	12.13 ± 0.49	8.36 ± 0.20	12.1 ± 0.53	23	–
*P. nigrum* (seeds)	*E-coli*	10.13 ± 0.28	9.3 ± 0.29	10.03 ± 0.37	22.16 ± 0.41	32	–
	*Salmonella*	10.33 ± 0.25	10.2 ± 0.24	10.16 ± 0.34	NIL	33	–
	*Shigella*	8.36 ± 0.32	7.2 ± 0.16	8.03 ± 0.41	NIL	31	–––
	*MRSA*	8.03 ± 0.45	8.13 ± 0.37	8.2 ± 0.16	16.2 ± 0.24	23	–
*C. cassia* (barks)	*E-coli*	10.23 ± 0.20	10.03 ± 0.38	9.06 ± 0.41	12.03 ± 0.45	32	–
	*Salmonella*	9.2 ± 0.24	11.13 ± 0.36	12.13 ± 0.28	10.43 ± 0.24	33	–
	*Shigella*	9.06 ± 0.32	10.26 ± 0.34	12.03 ± 0.36	10.13 ± 0.28	31	–––
	*MRSA*	8.03 ± 0.45	10.06 ± 0.41	10.13 ± 0.32	14.06 ± 0.32	23	–

NIL: No identified limit; Noted ANNOVA value (*p* < 0.01) for all samples.

**Table 6:** Minimum inhibitory concentration (MIC) of recipe 1 against different bacterial strains.

S. No	Bacteria	Methanol Extracts (mg/L)	Aqueous Extracts (mg/L)	Chloroform Extracts (mg/L)	n-Hexane Extracts (mg/L)
1	*E-coli*	11,000	12,500	14,000	15,000
2	*Salmonella*	12,000	11,500	13,000	14,000
3	*Shigella*	11,500	12,000	14,000	14,500
4	MRSA	14,000	14,500	15,000	15,000

**Table 7:** Minimum inhibitory concentration (MIC) of recipe 2 against different bacterial strains.

S. No	Bacteria	Methanol Extracts (mg/L)	Aqueous Extracts (mg/L)	Chloroform Extracts (mg/L)	n-Hexane Extracts (mg/L)
1	*E-coli*	12,000	11,000	13,000	14,000
2	*Salmonella*	10,500	12,000	12,500	13,000
3	*Shigella*	11,500	12,500	13,000	14,000
4	MRSA	15,000	14,000	15,000	15,000
